# On the potential value of eHTA: a commentary on “Defining Early Health Technology Assessment: Building Consensus Using Delphi Technique”

**DOI:** 10.1017/S0266462325100202

**Published:** 2025-07-21

**Authors:** Nick Dragojlovic, Mark Harrison, Larry David Lynd

**Affiliations:** 1 Faculty of Pharmaceutical Sciences, University of British Columbia, Vancouver, BC, Canada; 2 Centre for Advancing Health Outcomes, Providence Health Research Institute, Vancouver, BC, Canada

**Keywords:** early HTA, drug development, adoption risk, health technology innovation, research funding prioritization

## Abstract

The HTAi Health Technology Assessment (eHTA) Working Group’s (WG) development of a consensus definition of early eHTA, as reported in Grutters et al. (1), represents a major step towards the establishment of eHTA as a distinct subdiscipline of HTA. In a global landscape in which growth in pharmaceutical spending is driven by the increasing number of high-cost specialty drugs (2–6), and where the cost of new entrants is not systematically associated with their clinical benefit (7;8), broader uptake of eHTA by pharmaceutical innovators offers a route to improving the value delivered by our collective investments in drug research and development (R&D). As we argue in this commentary, the WG’s report provides a coherent framework within which to further define appropriate eHTA methods for specific use cases as well as eHTA’s relationship to other decision-making tools currently used by health technology innovators and funders.

The HTAi Health Technology Assessment (eHTA) Working Group’s (WG) development of a consensus definition of early eHTA, as reported in Grutters et al. (1), represents a major step towards the establishment of eHTA as a distinct subdiscipline of HTA. In a global landscape in which growth in pharmaceutical spending is driven by the increasing number of high-cost specialty drugs (2–6), and where the cost of new entrants is not systematically associated with their clinical benefit (7;8), broader uptake of eHTA by pharmaceutical innovators offers a route to improving the value delivered by our collective investments in drug research and development (R&D). As we argue in this commentary, the WG’s report provides a coherent framework within which to further define appropriate eHTA methods for specific use cases as well as eHTA’s relationship to other decision-making tools currently used by health technology innovators and funders.

Our focus on the role of eHTA in *pharmaceutical* innovation is shaped by our group’s experience developing the UBC eHTA Platform at the University of British Columbia’s Faculty of Pharmaceutical Sciences. Since 2021, we have been conducting eHTAs in collaboration with Canadian life science teams to assess the potential value of preclinical medical product candidates (9;10). In particular, our work has focused on developing eHTA study designs to inform the development and clinical translation of *platform biopharmaceutical technologies* (e.g., mRNA or siRNA delivery systems) at an early stage of development (i.e., still in an academic laboratory and/or at the spinoff stage), and where investigators have yet to invest significant resources in, or are considering a pivot away from their lead indication(s). Our goal is to help innovators select use cases for novel health technologies that have a high potential of being adopted and of significantly improving patient outcomes. In our experience, key barriers to applying eHTA in real-world settings include: (i) obtaining buy-in from funders and end-users (which requires a compelling value proposition for eHTA) and (ii) ensuring that investment in eHTA actually delivers value for money to stakeholders. As we argue below, the WG’s consensus definition for eHTA and the conceptual framework that underpins it help to clarify how these barriers to eHTA uptake may be overcome.

## Need for a differentiated value proposition for eHTA

Importantly, the consensus definition of eHTA crafted by the WG – “[a] health technology assessment conducted to inform decisions about subsequent development, research and/or investment by explicitly evaluating the potential value of a conceptual or actual health technology” (1) – helps to clarify both what eHTA *is* and what it *is not.* On the one hand, by highlighting the decision problems it is meant to address – R&D strategy and investment choices – it provides a clear differentiation from reimbursement-focused HTA without explicitly referencing the stage of development of the technology (“[t]he working group felt that the only clear distinction between early and other forms of HTA relates to the decision problems that the respective assessments are purposed to inform” (1)). Although the type of decision problem, the development stage, and the most appropriate eHTA methods are likely to be correlated in practice, we agree with this perspective and, by extension, that the primary decision makers are also distinct – namely, innovators, investors, funding agencies, and disease nonprofits for eHTA versus healthcare payers for reimbursement-focused HTA. However, from our direct experience of engaging with innovators as well as a survey we conducted to better understand the eHTA needs of the North American life science innovation community (11), it appears that only a minority have heard of eHTA, HTA, or even commonly used frameworks used to manage drug development such as target product profiles and bottom-up market sizing. Moreover, familiarity is especially low among academic life scientists, who collectively drive discovery and invention in this area.

### Mitigation of adoption risk is the key value proposition for eHTA

**Figure 1.**
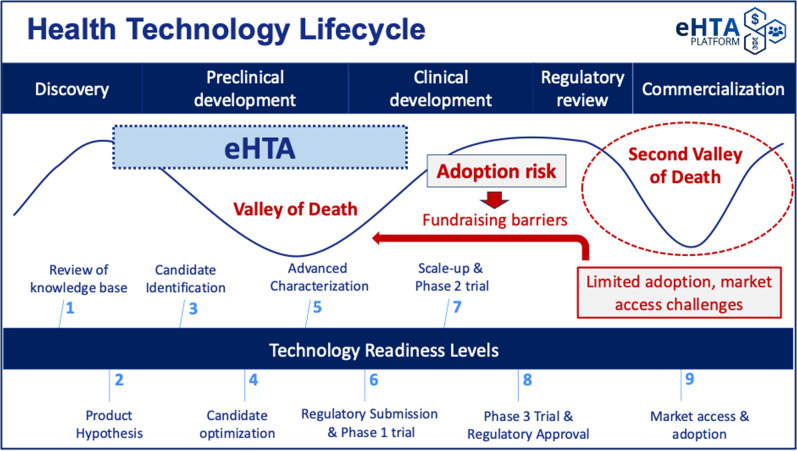
The key value proposition for eHTA is mitigation of adoption risk.

Low awareness of these concepts makes crafting a compelling value proposition for eHTA that resonates with potential academic end-users challenging, and although investors are likely to be more familiar with some of the methods used in eHTA, they may struggle to differentiate it from other analytic frameworks. Translational academic life scientists and early-stage university spin-offs are typically preoccupied with meeting scientific milestones for preclinical development, complying with regulatory requirements, and securing the funding necessary to move into clinical testing, rather than on the potential value of their product once approved. This is understandable because the primary focus of early-stage drug development is to avoid succumbing to the so-called (first) “Valley of Death” (12), in which scientific/technical barriers and/or fundraising difficulties prevent the completion of preclinical development for a product candidate (see Figure 1). In fact, the “target assessment frameworks” used by pharmaceutical companies to guide R&D (which are noted in the WG report) lay out a detailed approach to mitigating ***scientific risk*** through the structured evaluation of mode of action, disease linkage, safety, and technical feasibility (13). These types of systematic approaches to R&D decision making (14) have helped to mitigate biases (15) and contributed to the recent reversal in the historical decline in pharmaceutical R&D productivity (16). Notably, scientific risk is generally well understood by academic scientists with expertise in drug development or clinical translation.

However, these target assessment frameworks also recommend assessing several elements related to the ***commercial potential*** of a product candidate at an early stage, including the intellectual property (IP) landscape, unmet medical need, clinical differentiation, and market size (13;14;17–19). University technology transfer offices tend to emphasize IP protection and provide varying levels of support for market sizing, but typically do not conduct a systematic, evidence-based assessment of ***adoption risk*** (Figure 1). We use this term to refer specifically to the possibility of a medical product failing in the “second valley of death” (20) due to difficulties in securing timely reimbursement or adoption/market penetration. Adoption risk is currently particularly salient in the gene therapy space (20), with several recent examples of the commercial impact of slower-than-expected adoption. Bluebird Bio, a company previously valued at up to US$10 billion, was recently acquired for only US$30 million (21). Pfizer recently ended its partnership with Sangamo on a hemophilia A gene therapy despite positive Phase III results (21), a decision likely informed by BioMarin’s 2024 decision to narrow its commercial focus for Roctavian™ due to slow uptake (22). Finally, Pfizer has withdrawn Beqvez™ (a gene therapy for hemophilia B) from the market due to “weak demand from patients and doctors” (21).

These examples highlight the importance of unmet need and clinical differentiation, which feature in the pharmaceutical industry target assessment frameworks cited above. Specifically, the broad range of effective factor replacement products and other targeted therapies available for hemophilia A and B limited the clinical headroom – that is, the room for improving health outcomes – in those indications. However, they also point to the central role of HTA in reimbursement and market access in that gene therapies have struggled to demonstrate value to payers given high upfront prices and uncertainty about long-term clinical benefit (23), as well as to the important role of patient preferences in achieving adoption when therapeutic alternatives exist. These concepts fall firmly within the scope of HTA, and given that “[e]arly HTA is a subset of health technology assessment” (1), eHTA as defined by the WG is well suited to also assess adoption risk at an early stage of drug development.

As a result, eHTA can help to inform mitigation strategies for adoption risk, keeping in mind that one response can involve pivoting away from a specific lead indication or technology to another with greater potential value. This can also help to reduce the risk perceived by potential private sector investors whose willingness to invest is informed by the perceived commercial prospects for the therapeutic(s) under development and is critical in moving therapeutic product candidates past the first valley of death (Figure 1) (24–26). However, achieving these hypothetical impacts for eHTA will require highlighting both the problem (adoption risk) as well as the solution (eHTA) to early-stage innovators and funders, who tend to be much more familiar with, and almost exclusively focused on scientific risk and IP. At a system level, broad uptake of eHTA at very early stages of development would help translational life science teams in academia to focus their effort and resources on the clinical indications that are most likely to generate incremental value for patients and society and steer away from indications in which clinical and/or economic headroom is limited. However, this will require that the principles of eHTA and its value to innovators and to society be communicated in a way that is accessible to those in the broader health technology sector by clearly differentiating eHTA from, and connecting it to, existing tools used by life science stakeholders to assess commercial potential.

### Differentiating eHTA from other decision-making tools

In addition to clearly differentiating eHTA from early dialogue/early scientific advice and early awareness/horizon scanning (1), the WG’s report also implicitly provides guidance on how to distinguish eHTA from common frameworks currently used to evaluate commercial potential. Specifically, it notes that “[e]arly HTA is a subset of health technology assessment, which means that concepts from the main definition such as ‘in order to promote an equitable, efficient and high quality health system’ are implied and therefore not required in our core definition” (1). In short, *eHTA’s focus on health benefit* is the fundamental differentiator with existing tools used by industry and technology transfer offices, which tend to view decision-making through the prism of maximizing the *economic return for investors.* This distinction has several implications.

First, eHTA can be used to inform innovation by a broader range of innovators and funders, including those for whom profit maximization is not the primary motivation. For example, in addition to exploring commercial partnerships, a nonprofit drug development organization seeking to maximize the impact of its R&D activities could use eHTA to explore the potential population-level health benefit and budget impact of its innovations in order to prioritize drug candidates for which a strong business case could be made for *public funding of clinical trials.* This could be particularly relevant in the context of public health interventions with high potential value for society but lower commercial potential than other opportunities, or where the innovator cannot capture the value added by the health technology due to a lack of IP protection (e.g., drug repurposing, open science).

Second, eHTA can *complement existing analytic frameworks* used to guide R&D and investment decisions. For example, although the target assessment frameworks published by pharmaceutical companies include characterization of *unmet medical need* and *clinical differentiation* (which are predictive of potential health benefit and therefore aligned with an HTA value framework aimed at promoting a high-quality health system), they mostly do not provide guidance on which specific methods to conduct these assessments, and where explicit methods are outlined, they rely on key informant opinions which can themselves be vulnerable to group-think (19). In contrast, eHTA (which typically involves systematic synthesis of published evidence either in the form of targeted literature reviews or early cost-effectiveness models) can provide clear methodological guidance for generating unbiased, evidence-based assessments of these drivers of commercial potential.

Similarly, biotech asset and company *valuation methods* can be strengthened by the systematic evidence produced by eHTA. Key parameters for first-year or peak sales estimates for a drug candidate – such as disease prevalence, market share, market growth rate, and projected price (27) – can be informed by eHTA-generated evidence on treatable patient population, patient and provider preferences, and value-based pricing, which can enhance the defensibility of the sales forecasts and the valuations they inform (whether through the risk-adjusted net present value framework or the “venture capital” (VC) approach (27)). Even when using comparable company acquisition values to estimate the enterprise value (EV) of a startup at the projected exit date as part of a VC-style valuation, it is important to “use criteria that are… value-relevant” (Bogdan and Villiger (28), p. 297) to identify comparables, and eHTA-inspired indicators of potential value can be used to guide this process and the subsequent adjustment of the resulting EV estimates. This can be valuable for both innovators seeking investment and investors seeking high-value opportunities.

Finally, eHTA’s grounding in an HTA framework also helps to clarify when it might be most useful. On the one hand, for product classes like gene therapies for which new products are undergoing increasing levels of scrutiny by healthcare payers, eHTA can help to estimate how big the market could be *if HTA-informed value-based procurement is assumed* (e.g., by estimating the maximum reimbursable price using a headroom analysis (9) in which the novel product is assumed to be curative) or to *define the minimum target product profile (TPP) necessary to be cost-effective* in a given jurisdiction (29). On the other hand, eHTA is less applicable to contexts where uptake is likely to be a function of demand rather than need (e.g., direct-to-consumer medical products or services, such as some forms of genetic testing (30)).

## Ensuring eHTA delivers value to stakeholders

Ensuring that eHTA provides benefits commensurate with the resources invested in conducting it will ultimately be equally as important to long-term uptake as clarifying its value proposition. This will require customizing eHTA methods to specific decision problems, stakeholders, clinical areas, and technology types. As the WG reports, “[Delphi panelists] felt it would be useful to be explicit about several aspects of early HTA such as: who requests, carries out and pays for the HTA; what the outputs are; whether the process is confidential; and the role of the HTA agency” (1).

As argued above, using eHTA to provide evidence-based inputs for existing analytic frameworks and decision-making processes familiar to those stakeholders is likely to facilitate uptake and ensure that deliverables are useful to stakeholders. In addition to informing TPPs, market sizing, and valuation, eHTA could also be integrated into research priority-setting methodologies and research impact assessment frameworks used by funding agencies (31;32). Given that a key objective for public research funding agencies is to fund innovations with a high potential for improving health outcomes and health equity at the population level (33;34), eHTA is an obvious value framework to incorporate into health research funding processes. The form that eHTA takes in this context will depend on the values included in the granting agency or program’s impact assessment framework (e.g., the relative prioritization of foreseeable health benefits vs. scientific progress vs. economic benefits), as well as on whether the eHTA study in question involves assessing the potential value of a specific proposed technology or creating a TPP for a hypothetical technology to address a known health deficit (what the WG refers to as “technology-driven” vs. “needs-driven” eHTA (1)). In short, it is incumbent on eHTA practitioners to develop customized eHTA frameworks and tool kits for different use cases, and we agree with the WG that in doing so “it will be useful to relate early HTA to other fields of research such as bioethics, philosophy of technology, responsible research and innovation, and decision making under deep uncertainty” (1).

At the same time, what is also needed is a “meta-research” agenda to better understand the potential impact of eHTA and identify the use cases for which the return-on-investment is greatest. Existing evidence suggests that much public sector grant funding in the biomedical space is wasted, to a large extent due to research questions with low relevance and potential value to clinicians and patients (31;35); these same problems could afflict publicly funded eHTA if insufficient attention is paid to evaluating its impact. As the WG notes, however, “[m]uch early HTA… remains unpublished as it may be commercially sensitive” (1), which raises concerns about publication bias and limits the ability to assess the effectiveness of eHTA in improving innovation outcomes to date. However, studies like Grutters et al. (36) provide an excellent example of how program evaluations can be conducted for eHTA. It will be important for public funding bodies interested in supporting the use of eHTA to fund this type of meta-research as part of any eHTA program.

**Figure 2.**
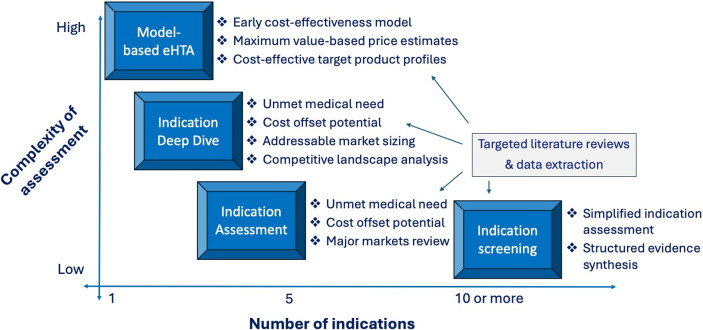
Trade-off between eHTA complexity and scope.

Finally, a related consideration that should be a topic of research and evaluation by the eHTA scholarly community is promoting the *resource efficiency* of the practice. The WG usefully identifies a wide variety of potentially appropriate eHTA methods at different stages of technology development (1), but an important area for future research will be to identify what the *minimal study complexity and scope* is in specific contexts to meet the informational needs of the decision makers (Figure 2). For example, in our experience, platform therapeutic technologies that are at an early stage of development (Technology Readiness Level 2–3) are unlikely to benefit from early economic modeling, because innovators are often evaluating a wide range of possible lead indications. Instead, a targeted literature review focused on key value drivers for each possible indication is likely to yield the evidence needed to inform a decision at a lower cost. In contrast, for a prototype diagnostic test targeted at a specific disease, economic modeling is likely to be crucial because a quantitative estimate of the potential downstream health impact of improved diagnosis will be a fundamental element of its value proposition (37). As such, considerations of operational efficiency should feature prominently in future eHTA meta-research.

## Conclusion

The HTAi eHTA WG’s report in this issue and the consensus definition for eHTA it outlines provide a solid foundation for developing eHTA both as a distinct subfield of HTA and as an innovation support practice that has a differentiated value proposition for stakeholders in the health technology innovation ecosystem. Moreover, because eHTA is positioned within HTA, it shares the underlying commitment to “promote an equitable, efficient and high quality health system,” and the development of eHTA on this basis and its broad uptake by the innovation community provides a plausible route through which to nudge health technology innovation toward use cases that will have the greatest benefit for patients and provide value for money to society.
